# Weighted Gene Coexpression Network Analysis of Features That Control Cancer Stem Cells Reveals Prognostic Biomarkers in Lung Adenocarcinoma

**DOI:** 10.3389/fgene.2020.00311

**Published:** 2020-04-22

**Authors:** Yi Liao, Yulei Wang, Mengqing Cheng, Chengliang Huang, Xianming Fan

**Affiliations:** Department of Respiratory and Critical Care Medicine II, The Affiliated Hospital of Southwest Medical University, Luzhou, China

**Keywords:** lung adenocarcinoma, cancer cell stemness, WGCNA, TCGA, prognosis

## Abstract

**Purpose** We aimed to identify new prognostic biomarkers of lung adenocarcinoma (LUAD) based on cancer stem cell theory.

**Materials and Methods:** RNA-seq and microarray data were obtained with clinical information downloaded from The Cancer Genome Atlas (TCGA) and the Gene Expression Omnibus (GEO) databases. Weighted gene coexpression network analysis (WGCNA) was applied to identify significant module and hub genes. The hub genes were validated via microarray data from GEO, and a prognostic signature with prognostic hub genes was constructed.

**Results** LUAD patients enrolled from TCGA had a higher mRNA expression-based stemness index (mRNAsi) in tumor tissue than in adjacent normal tissue. Some clinical features and prognoses were found to be highly correlated with mRNAsi. WGCNA found that the green module and blue module were the most significant modules related to mRNAsi; 50 key genes were identified in the green module and were enriched mostly in the cell cycle, chromosome segregation, chromosomal region and microtubule binding. Six hub genes were revealed through the protein-protein interaction (PPI) network and Molecular Complex Detection (MCODE) plugin of Cytoscape software. Based on external verification with the GEO database, these six genes are not only expressed at different levels in LUAD and normal tissues but also associated with different clinical features. In addition, the construction of a prognostic signature with three hub genes showed high predictive value.

**Conclusion** mRNAsi-related biomarkers may suggest a new potential treatment strategy for LUAD.

## Introduction

Lung cancer remains the leading malignancy in terms of morbidity and mortality according to the latest large-scale epidemiological survey of 20 regions on five continents, and lung cancer incidence (31.5%) and mortality (27.1%) are the highest in men from both developed and developing countries (Bray et al., 2018). Moreover, a survey from China showed that both worldwide and in China, the cancer type with the highest total incidence and mortality is lung cancer, which accounts for 11.6% (global) and 20% (China) of total cancer-related morbidity and 18.4% (global) and 27.3% (China) of total cancer-related mortality (Chen et al., 2018). Among the pathological types of lung cancer, non-small-cell lung cancer (NSCLC) accounts for the majority of cases (approximately 80%), and lung adenocarcinoma (LUAD) is one of the most common types (Barlesi et al., 2016).

There is still no definite conclusion about the origin of LUAD and its pathological mechanism. However, an increasing number of studies have shown that tumor stem cells are valuable in research and play an important role in tumor differentiation, metastasis and drug resistance (Friedmann-Morvinski and Verma, 2014; Leon et al., 2016; Shibue and Weinberg, 2017). Based on these theories, Malta et al. (2018) proposed a new concept – the stemness index – to measure tumor development and evaluate the reliability of stem cell indices for analyzing tumors using TCGA data. These researchers calculated the mRNA expression-based stemness index (mRNAsi) and epigenetically regulated-mRNAsi (EREG-mRNAsi) using TCGA data. The mRNAsi is calculated based on expression data and ranges from 0 to 1, with values closer to 1 indicating low differentiation and strong stem cell characteristics. This previous study also confirmed that the stem cell index is related to clinical and molecular cancer typing, biological processes, cancer metastasis, intratumoral heterogeneity, and the immune microenvironment, providing new ideas and strategies for cancer research (Malta et al., 2018).

Weighted gene coexpression network analysis (WGCNA) is a systematic biological method used to describe the patterns of gene associations between different samples. This method can identify candidate biomarker genes or therapeutic targets based on the interconnectivity of gene sets and the association between gene sets and phenotypes (Langfelder and Horvath, 2008). Instead of focusing on only differentially expressed genes (DEGs), WGCNA uses information from thousands of the most varied genes or all the genes to identify gene sets of interest and identifies significant associations with phenotypes. By converting thousands of genes associated with phenotypes into dozens of gene sets associated with phenotypes, the problem of multiple hypothesis testing and correction is eliminated. Moreover, according to the methods and principles of WGCNA and miRNA, some scholars have found hub genes related to bladder cancer (Pan et al., 2019).

Considering the strong association between tumor stem cells and tumor pathogenesis, this study aimed to identify target genes related to the regulation of LUAD stem cells. We used WGCNA to analyze high-throughput sequencing data from relevant public databases, obtained the module with the highest correlation with mRNAsi and identified relevant biomarkers. Another data set generated from multiple chips was used to verify the correlations between these biomarkers and the clinical characteristics of LUAD. In addition, the prognostic ability of these biomarkers was examined.

## Materials and Methods

### Data Processing

We downloaded the RNA-seq data and clinical information for LUAD from the TCGA database^1^. Then, we converted Ensembl IDs to gene names using the Ensembl database^2^ and performed log2 processing of the data. Malta et al. (2018) used an innovative single-class logistic regression machine learning algorithm to extract sets of transcriptome and epigenetic characteristics from non-transformed pluripotent stem cells and their differentiated progeny in TCGA. Moreover, we can download the data for the calculated mRNAsi belonging to the TCGA database. Therefore, we downloaded the calculated mRNAsi and EREG-mRNAsi of each LUAD patient from https://www.ncbi.nlm.nih.gov/pmc/articles/PMC5902191/ (Malta et al., 2018). Any LUAD sample from the TCGA database with incomplete clinical patient information was excluded.

To verify the connection between the stem cell index and clinical characteristics, we obtained corresponding data from the GEO database. The inclusion criteria were as follows: (1) the sample size of the GEO data set was greater than 100, and the research focus was human LUAD; (2) each sample had adequate clinical information; (3) all data sets contained the corresponding hub genes identified for validation; and (4) a platform was available to convert probe names into gene names. After defining the gene set according to the inclusion criteria, we downloaded the series matrix files and platform from the GEO database and transformed the probe names into gene names.

### Correlations of mRNAsi With Clinical Characteristics

We studied the significant differences in mRNAsi between normal and LUAD tissues and between patients with and without recurrence by the unpaired t test with GraphPad Prism version 7 (64 bit). Since postoperative adjuvant therapy may affect tumor recurrence (Kaplan et al., 2016), we did not include patients who received radiotherapy or chemotherapy when comparing recurrent and non-recurrent patients. One-way ANOVA was used to compare mRNAsi differences between groups of patients based on TNM stage.

For prognostic comparisons, we compared the overall survival (OS) and progression-free survival (PFS) rates. OS is defined as the time between tumor diagnosis and death (from any cause), and PFS is defined as the time between tumor diagnosis and disease progression (in any way) or death (due to any reason). We used X-tile software to identify the best cutoff value and divided the cohort into high and low groups based on the mRNAsi value relative to the cutoff. The working principle of X-tile is to conduct a statistical analysis by grouping different values as truncation values. The result with the smallest *P*-value can be considered the best truncation value, and the optimal truncation value for survival data can be quickly found (Camp et al., 2004). We generated survival curves using Kaplan-Meier analysis and calculated the *P*-value by the log rank test for two groups in the survminer package of R software (v 3.6.0). *P* < 0.05 was considered to indicate a significant difference.

### Differentially Expressed Genes (DEGs)

The limma package (Ritchie et al., 2015) was used to identify genes that differ between LUAD and normal tissues. For genes with multiple probes, we averaged the values. Genes with a log_2_ fold-change (FC) > 1 and an adjusted *P* < 0.05 were considered DEGs.

### WGCNA

#### Construction of a Coexpression Network

The WGCNA package (Langfelder and Horvath, 2008) was used to construct a coexpression network. The goodSamplesGenes function was used to remove genes with large deletions and outliers after building the sampleTree. Pearson correlation coefficients between each group of genes were also calculated, and their absolute values were used to construct the gene expression similarity matrix: the formula for that is Eq. 1, where *x*_*i*_ and *x*_*j*_ are the nodes *i* and *j* of the network. The best β value was selected to build the proximity matrix so that our gene distribution conformed to a scale-free network based on connectivity. The adjacent and topological matrices were obtained from the β values. The obtained topological overlap matrix (TOM) was clustered by dissimilarity between genes, and in Eq. 2, *L*_*ij*_ represents the sum of the product of the adjacency coefficients of the nodes joined by gene i and gene j. K represents the sum of the adjacency coefficients of all nodes connected individually by the gene. Then, the trees were divided into different modules by the dynamic shear method (the minimum number of genes in each module was 50). We incorporated all DEGs into the coexpression network after excluding outlier samples. β = 3 met the soft-threshold parameters of the construction requirements for a scale-free distribution, and the curve reached R2 = 0.97. MEDissThres was set to 0.7 to merge similar modules.

(1) a=i⁢j|cor(x,ix)j|β

(2) $$T\,O\,M=\frac{1_{i\,j}+a_{i\,j}}{\min\left({\text{k}}_{i},{\text{k}}_{j}\right)+1-a_{i\,j}}$$

#### Identification of Significant Modules

We selected the hierarchical clustering module closely related to mRNAsi and EREG-mRNAsi as the module for subsequent analysis. Genetic significance (GS), module significance (MS), and module eigengenes (MEs) were calculated. GS is defined as the level of correlation between gene expression and clinical information and is calculated by log10 transformation of the *P*-value in the linear regression. MS is the average of the significance of all genes in the module. ME is the first principal component obtained by principal component analysis of the gene expression matrix of each module. In addition, to clarify the relationship between modules and the immune landscape, we adopted a single simple gene set enrichment analysis (ssGSEA) method by the GSVA package in R. The analysis of 28 types of immune infiltrating cells (TIICs) in tumor tissues by this algorithm depends on the specific labeled genomes of immune cells in each subgroup. Through this algorithm, we can obtain corresponding scores to reflect the TIICs infiltration abundance of a single sample. Among all the modules, the one with the highest absolute MS value was considered to be related to clinical characteristics (mRNAsi, EREG-mRNAsi, and ssGSEA scores); this module deserves further discussion.

#### Identification of Key Genes

After identifying significant modules, we calculated the GS and module membership (MM, correlation between the module’s own genes and gene expression profiles) for each key gene. MM is used to describe the degree of association between nodes in a particular module and other nodes in the module, that is, the degree of internal connectivity of the module. To further identify genes related to the trait of mRNAsi, we selected genes with MM > 0.8 and cor. gene GS > 0.6 as key genes.

### Functional Enrichment

The clusterProfiler package (Yu et al., 2012) was used to perform functional enrichment for Gene Ontology (GO) and Kyoto Encyclopedia of Genes and Genomes (KEGG); the GO categories were biological process (BP), cellular component (CC), and molecular function (MF). The threshold was set at adjusted *P* < 0.05. Functional enrichment analysis was used for significant modules and key genes obtained by WGCNA.

### Protein-Protein Interaction (PPI) Network and Hub Gene Identification

We used key genes identified by coexpression network analysis to build PPI networks using the String database^3^. The String database searches for known and predicted protein interactions and studies the interaction networks between proteins to help identify core regulatory genes. The inclusion criteria of the hub genes are as follows: the genes with the highest MCODE_Score performed by screening with MCODE (Saito et al., 2012) with a default parameter setting that is degree cut-off = 2, node score cut-off = 0.2 and K-core value = 2 by Cytoscape (version 3.6.1; 64-bit; www.cytoscape.org/) (Smoot et al., 2011). We also calculated coexpression relationships among key genes based on the gene expression levels to determine their strength at the transcriptional level. The Pearson correlation between genes was calculated using the R corrplot package.

### Validation of Hub Genes

To further verify the connection between the hub genes and clinical characteristics, we analyzed corresponding data from the GEO database for verification. The inclusion criteria for the qualified samples of GEO database were as follows: (1). The samples were belong to human LUAD or human normal tissue. (2). each sample had adequate clinical information. (3). The sample all contain the corresponding hub genes for validation. After defining the gene set according to the inclusion criteria, we downloaded the series matrix files and platform from the GEO database and transformed the probe name into the gene name. An unpaired t test was used to compare two groups, and comparisons among multiple groups were performed with one-way ANOVA. To analyze the correlation of TIICs with each hub gene, we used the TIMER^4^ online database. It also uses RNA-seq expression profile data to detect the infiltration of immune cells in tumor tissues. Moreover, TIMER provided infiltration of six types of immune cells (B cells, CD4 + T cells, CD8 + T cells, neutrophils, lymphocytes and dendritic cells).

### Survival Analysis

#### Establishment of a Risk Assessment Model

A multivariate Cox proportional hazards regression analysis was carried out for hub genes significantly associated with OS in univariate proportional hazards regression analysis to further identify independent hub genes with the best prognostic efficacy using the Akaike information criterion (Yamaoka et al., 1978). A risk score formula was created using the hub genes that *P* < 0.05 obtained through multivariate Cox proportional hazards regression analyses. In Eq. 3, *n* denotes the number of prognostic hub genes, Gi represents the expression value of the *i*th hub genes, and weight i denotes the coefficient of each significant hub gene. With the median risk score as the dividing line, we divided the patients into high-risk (>median risk score) and low-risk (<median risk score) groups, and the Kaplan–Meier method was used to estimate the survival of high-risk and low-risk patients. To validate the effect of the risk assessment model, we used a time-dependent receiver operating characteristic (ROC) curve for verification.

(3) $$\mathrm{Risk}\,\mathrm{score}=\sum_{i=1}^{n}G_{i}\times {\text{weight}}_{i}$$

#### Construction of a Nomogram

Univariate and multivariate Cox regression analyses were performed for clinical factors and risk scores, and factors with *P* < 0.05 were considered to be independent prognostic factor and used to construct the nomogram with a 1-, 3-, and 5-year survival rate using the R rms package (Frank and Harrel, 2019). To verify the accuracy of the nomogram in predicting patient survival, we used a calibration, time-dependent ROC curve and Harrell’s C statistics (Heagerty and Zheng, 2005).

## Results

### The Correlation of mRNAsi and Clinical Characteristics in LUAD

**FIGURE 1:**
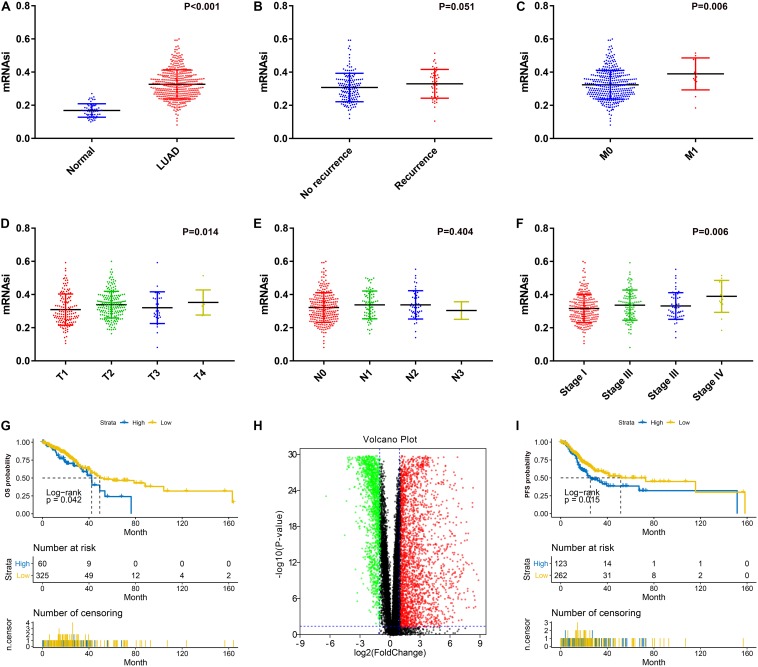
**(A)** Differences in mRNAsi between normal (57 samples) and LUAD (385 samples) tissues. **(B)** Differences in mRNAsi between LUAD patients without recurrence (154 samples) and with recurrence (46 samples) after primary treatment without adjuvant therapy. **(C)** Differences in mRNAsi between LUAD patients with M0 (370 samples) and M1 (15 samples) stage. **(D)** Comparison of mRNAsi in four different T stages (T1, 124 samples; T2, 75 samples; T3, 32 samples; T4, 9 samples). **(E)** Comparison of mRNAsi in four different N stages (N0, 260 samples; N1, 75 samples; N2, 48 samples; N3, 2 samples). **(F)** Comparison of mRNAsi in four different AJCC stages (Stage I, 215 samples; Stage II, 99 samples; Stage III, 56 samples; Stage IV, 15 samples). **(G)** Kaplan–Meier curves show that the low mRNAsi group had a better prognosis than the high mRNAsi group for OS. **(H)** Volcano map of differentially expressed genes; green indicates downregulated genes, and red indicates upregulated genes. **(I)** Kaplan–Meier curves show that the low mRNAsi group had a better prognosis than the high mRNAsi group for PFS. LUAD, lung adenocarcinoma; mRNAsi, mRNA expression-based stemness index; AJCC, American Joint Committee on Cancer; OS, overall survival; PFS, progression-free survival.

After excluding unqualified samples from the TCGA database (Table 1), we compared the mRNAsi with the clinical characteristics. As shown in Figure 1A, a significant difference between the mRNAsi values of the LUAD tissues and normal tissues was observed. The mRNAsi of tumor tissues was higher than that of normal tissues. Significant differences were also found for T stage (Figure 1D), M stage (Figure 1C) and AJCC stage (Figure 1F). There were significant differences between the T1 and T2 stages (*P* = 0.010), stage I and stage IV (*P* = 0.009). However, there was no significant difference in the mRNAsi values between the relapse groups (Figure 1B) and N stages (Figure 1E). Furthermore, there was no significant difference in pairwise comparisons of the N stages. In terms of prognosis, LUAD patients with a high mRNAsi showed significantly worse outcomes than those with a low mRNAsi for both OS and PFS (Figures 1G,I).

**TABLE 1:** Basic characteristics of the gene expression profile data. LUAD, lung adenocarcinoma.

**Record**	**Platform**	**Normal**	**LUAD**	**Number of excluded samples**	**Country**	**Year**
TCGA	IIIumina HiSeq	57	385	134	United States	2018
GSE13213	GPL6480 Agilent-014850 Whole Human Genome Microarray 4 × 44K G4112F (Probe Name version)	0	117	0	Japan	2009
GSE31210	GPL570 [HG-U133_Plus_2] Affymetrix Human Genome U133 Plus 2.0 Array	0	226	0	Japan	2011
GSE26939	GPL9053 Agilent-UNC-custom-4 × 44K	0	116	0	United States	2012
GSE32867	GPL6884 Illumina HumanWG-6 v3.0 expression beadchip	58	58	0	United States	2012
GSE41271	GPL6884 Illumina HumanWG-6 v3.0 expression beadchip	0	182	93	United States	2013
GSE43458	GPL6244 [HuGene-1_0-st] Affymetrix Human Gene 1.0 ST Array [transcript (gene) version]	30	80	0	United States	2013

### Screening of DEGs

There were significant differences between mRNA levels in normal tissues and LUAD tissues; thus, we identified DEGs based on the comparison between the two groups. After normalization and log2 processing of the data, we found a total of 4340 DEGs, including 2571 upregulated genes and 1769 downregulated genes. The volcano map is shown in Figure 1H.

### Identification of the Most Significant Modules and Key Genes

**FIGURE 2:**
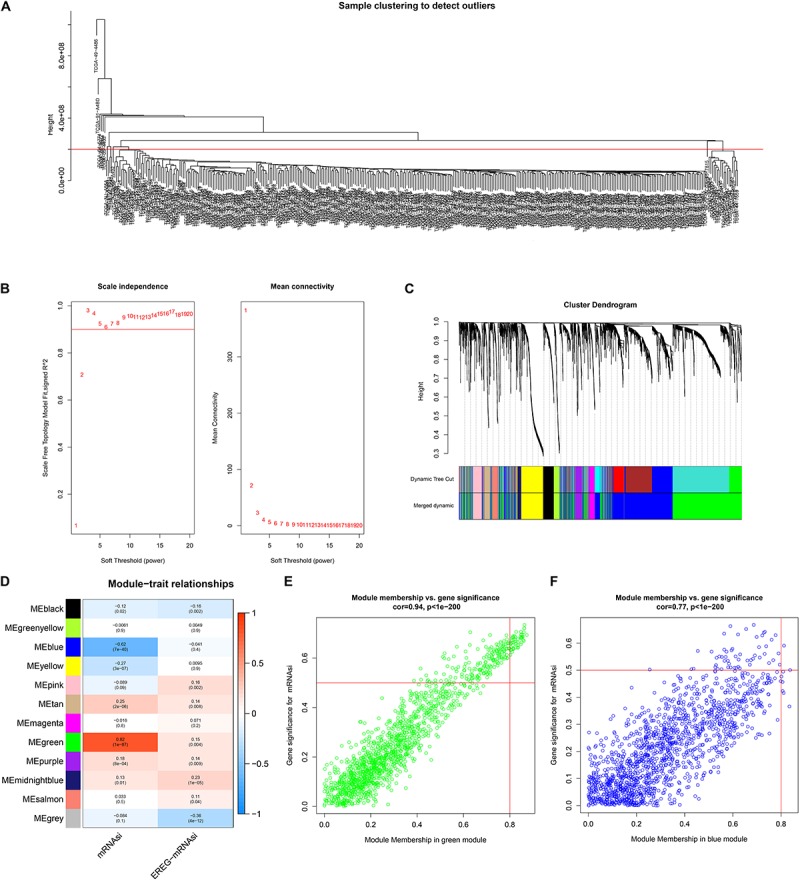
Weighted gene coexpression network of LUAD. **(A)** Clustering of samples and removal of outliers. **(B)** Analysis of network topology for various soft-thresholding powers. The left panel shows the scale-free fit index, signed *R*^2^ (*y*-axis) and the soft threshold power (*x*-axis). β = 3 was chosen for the subsequent analysis. The right panel shows that the mean connectivity (*y*-axis) is a strictly decreasing function of the power β (*x*-axis). **(C)** The cluster dendrogram of genes of LUAD patients. Each branch in the figure represents one gene, and every color below represents one coexpression module. **(D)** Correlation between the gene module and clinical characteristics, including mRNAsi and EREG-mRNAsi. The correlation coefficient in each cell represented the correlation between the gene module and the clinical characteristics, which decreased in size from red to blue. **(E)** Scatter diagram for module membership vs. gene significance for mRNAsi in the green module. **(F)** Scatter diagram for module membership vs. gene significance for mRNAsi in the blue module. LUAD, lung adenocarcinoma; mRNAsi, mRNA expression-based stemness index; EREG, epigenetically regulated.

The best soft-threshold parameters and the scale-free distribution are shown in Figure 2A. Finally, we obtained 15 modules (Figure 2B). The green (*R*^2^ = 0.82, *P* = 1e-87) and blue (*R*^2^ = −0.62, *P* = 7e-40) modules were found to be associated with the mRNAsi of LUAD (Figure 2C). In addition, the genes in the green (cor = 0.94, *P* < 1e-200) and blue modules (cor = 0.77, *P* = 1e-200) showed high GS and MM based on an intramodular analysis (Figures 2D,E). In addition, the green module has a negative correlation with ssGSEA scores, while the blue module has a positive correlation (Supplementary Figure S1). The green module was selected for subsequent research due to the highest positive correlation with mRNAsi. The 51 key genes that are MM > 0.8 and cor. gene GS > 0.6 were considered key genes for the green module.

### Functional Enrichment of Significant Modules and Key Genes

**FIGURE 3:**
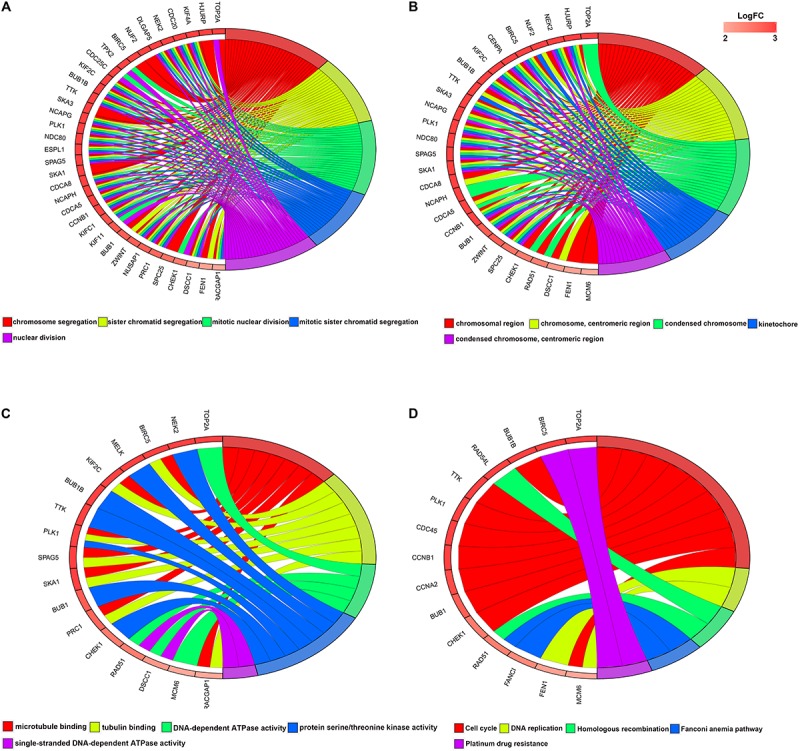
Circular plot of LUAD DEG enrichment analysis. **(A)** Biological processes, **(B)** molecular function (MF), and **(C)** cellular component. **(D)** KEGG pathway enrichment analysis. DEGs, differentially expressed genes; KEGG, Kyoto Encyclopedia of Genes and Genomes.

For the significant modules and key genes, GO and KEGG pathway enrichment analyses were performed. The top five enriched results are presented in Supplementary Figure S2 for green and blue modules. Figure 3 shows 51 key genes. These key genes are mostly enriched in chromosome segregation, chromosomal region and microtubule binding, and these findings are strongly related to the cell cycle.

### PPI Network and Hub Gene Identification

**FIGURE 4:**
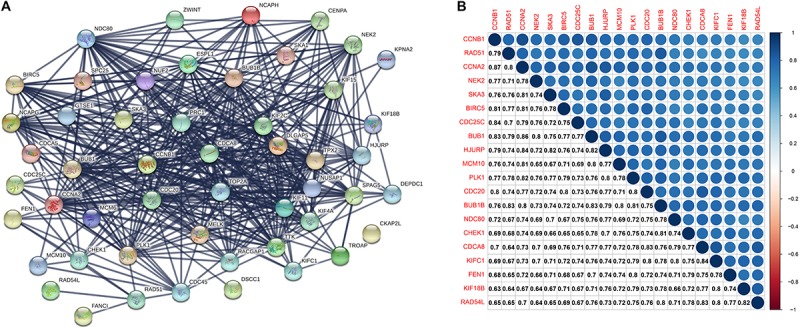
**(A)** The protein-protein interaction between key genes. The thickness of the solid line represents the strength of the relationship. **(B)** Correlation between the top 20 key genes according to MCODE scores at the transcriptional level. MCODE, Molecular Complex Detection.

A PPI network consists of 51 nodes and 1212 edges (Figure 4A). The recombination protein RAD54-like (*RAD54L*) is the seed node judged by the MCODE plugin of Cytoscape software, and the following genes with MCODE scores = 40 and nodes > 40 degrees will be considered hub genes: checkpoint kinase 1 (*CHEK1*), recombination protein A 51 (*RAD51*), kinesin family member 18B (*KIF18B*), kinesin family member C1 (*KIFC1*) and flap structure-specific endonuclease 1 (*FEN1*). Furthermore, the six hub genes were significantly correlated with each other (Figure 4B).

### Validation of Hub Gene Expression

**FIGURE 5:**
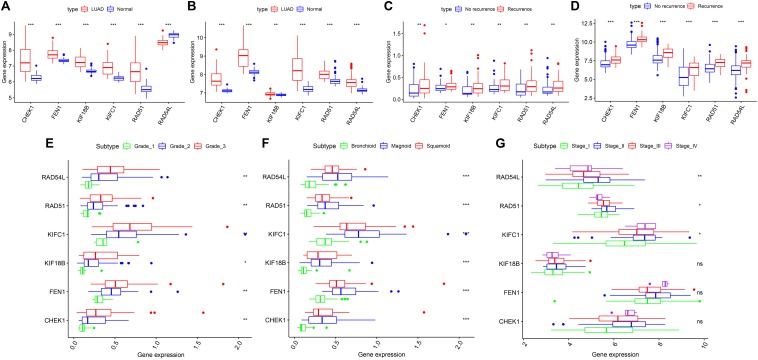
The six hub genes were verified in the GEO database. **(A)** In GSE43458, the expression of the five hub genes was higher in LUAD than in normal tissue, except *RAD54L*. **(B)** In GSE32867, the expression of the six hub genes was higher in LUAD than in normal tissue. **(C)** In GSE13213, the expression of the six hub genes was higher in the LUAD patients with recurrence than in those without recurrence. **(D)** In GSE31210, the expression of the six hub genes was higher in the LUAD patients with recurrence than in those without recurrence. **(E)** In GSE26939, statistically significant differences existed in six hub genes of different grades. **(F)** In GSE26939, statistically significant differences exist in six hub genes of different molecular subtypes. **(G)** In GSE41271, *RAD54L*, *RAD51* and *KIFC1* were significantly differentially expressed in different AJCC grades. LUAD, lung adenocarcinoma; AJCC, American Joint Committee on Cancer. *means *P* < 0.05, **means *P* < 0.01, ***means *P* < 0.001.

According to our inclusion criteria, we analyzed six chips (Table 1 and Supplementary Table S1) that contain qualified samples from the GEO database, namely, GSE13213 (Tomida et al., 2009), GSE31210 (Okayama et al., 2012; Yamauchi et al., 2012), GSE26939 (Wilkerson et al., 2012), GSE32867 (Selamat et al., 2012), GSE41271 (Sato et al., 2013; Riquelme et al., 2014; Girard et al., 2016; Parra et al., 2016), and GSE43458 (Kabbout et al., 2013). In GSE41271, 93 samples were excluded because they belonged to LUSC. There were significant differences in the expression of the six hub genes in both chips (GSE32867, GSE43458). However, *RAD54L* was inconsistently expressed between LUAD and normal tissues, and the remaining five genes were highly expressed in LUAD tissues compared to normal tissues (Figures 5A,B). The patients with LUAD in GSE31210 and GSE13213 did not receive any adjuvant radiotherapy or chemotherapy after the operation. We found that the six hub genes were highly expressed in patients with tumor recurrence (Figures 5C,D). For GSE26939, we found that there were significant differences in the expression of the six hub genes in LUAD patients with different grades, and with the increase in staging, the gene expression also increased (Figure 5E). However, for different AJCC grades, only *RAD54L*, *RAD51*, and *KIFC1* were significantly expressed with regard to different grades (Figure 5G). Notably, in LUAD molecular subtypes (bronchioid, magnoid, and squamoid), 6 of the hub genes showed differences in expression. Magnoid subtype expression was the highest and lowest compared to bronchioid subtype expression (Figure 5F). As shown in Supplementary Figure S3, six hub genes were all moderately negatively correlated with B cells, and all moderately negatively correlated macrophages except *CHEK1*. As the expression of these genes increased, the amount of TIICs decreased, while *RAD54L* was positively correlated with CD4 + T cells. High expression of this immune cell also increased.

### Survival Analysis

#### Establishment of a Risk Assessment Model

**FIGURE 6:**
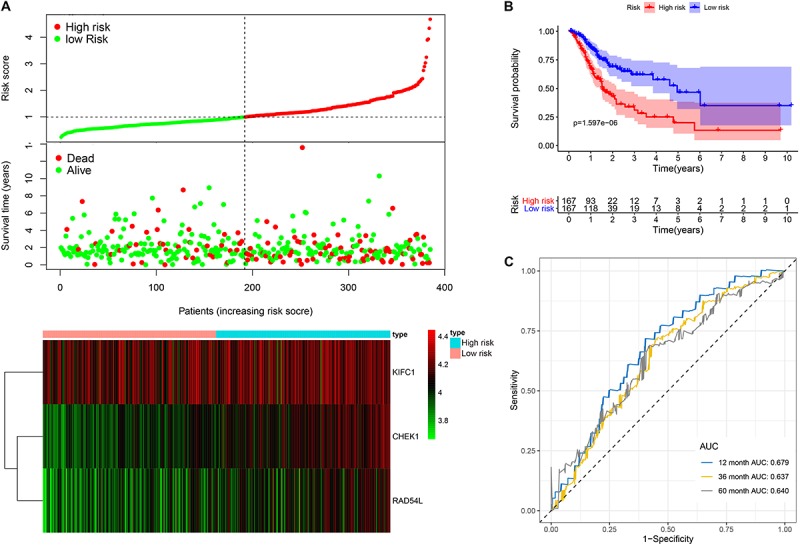
The three-hub mRNA signature in the prognosis of OS of LUAD patients. **(A)** The distribution, patient survival status and heatmap of the three-hub mRNA expression profiles. **(B)** Kaplan–Meier survival estimates OS of LUAD patients according to the three-hub mRNA signature. **(C)** ROC analysis for OS prediction within 1–, 3–, and 5–year as the defining point of the three-hub mRNA signature. LUAD, lung adenocarcinoma; ROC, receiver operating characteristic; OS, overall survival.

This model consists of three prognostic hub genes that are independent risk factors and were used with Eq. 3 to calculate the risk scores (Table 2). The risk score is (0.411^∗^ expression level of *CHEK1*) + (−0.367^∗^ expression level of *KIFC1*) + (0.326^∗^ expression level of *RAD54L*). The risk score distribution with the survival status of all included LUAD patients from TCGA is shown in Figure 6A. Kaplan-Meier analysis showed that LUAD patients with high risk scores had significantly shorter OS times than patients with low risk scores (Figure 6B). Time-dependent ROC analysis showed that the risk assessment model had good predictive performance for the 1-, 3-, and 5-year predictive probability (Figure 6C).

**TABLE 2:** Univariable and multivariable Cox regression analysis of the three-hub gene signature.

**Genes**	**Univariate analysis**			**Multivariate analysis**			**Coefficients**
	**HR**	**95%CI**	***P*-value**	**HR**	**95%CI**	***P*-value**	
CHEK1	1.361	1.149–1.613	<0.001	1.490	1.131–1.963	0.005	0.411
RAD51	1.243	1.049–1.474	0.012	1.018	0.712–1.454	0.523	–
KIF18B	1.117	1.074–1.280	0.016	1.008	0.753–1.349	0.058	–
KIFC1	0.775	0.426–0.848	0.042	0.662	0.460–0.952	0.026	−0.367
FEN1	1.224	1.079–1.531	0.012	1.116	0.649–1.921	0.691	–
RAD54L	1.187	1.024–1.375	0.023	1.338	1.011–1.771	0.042	0.326

#### Construction of a Nomogram

**FIGURE 7:**
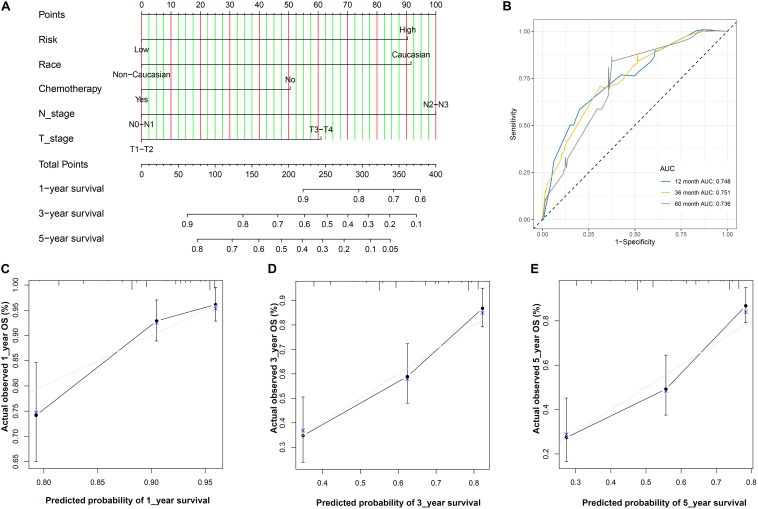
**(A)** The nomogram for predicting probabilities of patients with 1–, 3–, and 5–year OS. **(B)** ROC curve based on the nomogram for 1–, 3–, and 5–year OS probability. **(C)** The calibration plots for predicting patient 1–year OS. Nomogram–predicted probability of survival is plotted on the *x*–axis; actual survival is plotted on the *y*–axis. **(D)** The calibration plots for predicting patient 3–year OS. **(E)**. The calibration plots for predicting patient 5–year OS. LUAD, lung adenocarcinoma; ROC, receiver operating characteristic; OS, overall survival.

After Cox regression analysis combined with the clinical information, we found that the risk score was still a significant risk factor affecting prognosis (Table 3). Because there were relatively few patients in the N3 and T4 stages, we set the T\N stage as a binary variable (T1–T2/T3–T4 and N1–N2/N3–N4) to facilitate subsequent analysis. The nomogram contains five prognostic risk factors, namely, race, T stage, N stage, chemotherapy and risk score (Figure 7A). The C index is 0.724, and the ROC curves (Figure 7B) and calibration curve (Figure 7C) of the 1-, 3-, and 5-year OS all indicate that our model has good predictive ability.

**TABLE 3:** Univariable and multivariable Cox regression analysis of patient clinical characteristics.

**Variable**	**Univariate analysis**			**Multivariate analysis**		
	**HR**	**95%CI**	***P*-value**	**HR**	**95%CI**	***P*-value**
Age	1.010	0.991–1.030	0.296	–		
Sex (female/male)	0.670	0.633–1.342	0.921	–		
Race (Caucasian/non-Caucasian)	2.330	1.246–4.355	0.008	2.327	1.222–4.429	0.016
Smoke history (no/yes)	0.671	0.337–1.335	0.256	–		
T stage (T1-T2/T3-T4)	1.449	1.134–1.851	0.003	1.241	1.032–1.492	0.021
N stage (N1-N2/N3-N4)	1.709	1.367–2.136	<0.001	2.800	1.796–4.366	<0.001
M stage (M0/M1)	1.654	0.804–3.403	0.172	–		
Chemotherapy (No/Yes)	0.780	0.065–0.098	0.041	0.634	0.410–0.980	0.040
Radiotherapy (No/Yes)	2.025	1.310–3.130	0.001	1.369	0.828–2.265	0.221
Risk score (Low/High)	2.707	1.782–4.112	<0.001	2.533	1.645–3.901	<0.001

## Discussion

NSCLC is the king of cancers due to its extremely high mortality and morbidity, but its pathogenesis is still unclear. However, an increasing number of studies have found that cancer stem cells (CSCs) play an important role in the development and drug resistance of NSCLC (Herreros-Pomares et al., 2019; Huang et al., 2019). In this study, we identified the significance of the mRNAsi in the clinical characteristics of patients with LUAD with the help of the TCGA database and the mRNAsi corresponding to each sample. At the same time, hub genes related to the mRNAsi were obtained by the WGCNA method and verified with external data from the GEO database. The results also indicated that these 6 genes are important factors in clinical characteristics and that these genes are highly correlated with each other. Finally, after adjusting for possible prognostic factors, we obtained a predictive model containing three prognostic genes with good predictive power.

We used WGCNA to obtain a green module of interest related to the mRNAsi, and functional enrichment analysis suggested that most of the gene functions were enriched in the cell cycle and DNA replication pathway. GO analysis also indicated that most of the functions of this module are enriched in chromosome segregation, chromosomal region, and microtubule binding. These pathways were confirmed to be related to the occurrence, development and drug resistance of NSCLC (Liu et al., 2019; Sun et al., 2019). These functional pathways are similar to the biological functions of CSCs (Wang et al., 2019). The hub genes identified by WGCNA may serve as new therapeutic targets or biomarkers for LUAD research.

*CHEK1* plays a central role in DNA damage. *CHEK1* also regulates the cell cycle, coordinates cellular activities, and participates in DNA repair. *CHEK1* is highly expressed in breast cancer, colon cancer, liver cancer, gastric cancer and other tumors, and *CHEK1*-related signaling pathways have been confirmed in a wide variety of tumors. *CHEK1* is regarded as a target gene for potential tumor treatment (McNeely et al., 2014). In addition, Doerr et al. (2017) confirmed that *CHEK1* is highly expressed in small-cell lung cancer (SCLC) compared with LUAD. *In vivo* studies in mice showed that blocking the *CHEK1*-related pathway can induce genotoxic damage and apoptosis in SCLC cells but not in LUAD (Doerr et al., 2017). However, Yu et al. (2012) demonstrated in mice that miR-195 regulates the response of NSCLC cells to microtubule-targeting agents (MTAs) by targeting *CHEK1*. The overexpression of *CHEK1* contributes to the development of resistance to MTAs, while the knockout of *CHEK1* contributes to the enhancement of MTAs and inhibition of the growth of NSCLC cells.

*RAD51* is also highly expressed in many cancers (Lee et al., 2019; Zhang W. et al., 2019) and has been identified as a radiosensitive target for many cancers (Chen et al., 2012). Sanada et al. (2019) evaluated the *miR-143-5p* molecular network in LUAD using whole-genome sequencing combined with miRNA database analysis and identified 11 prognostic target genes, including *RAD51*. Another study showed that the abnormal expression of other genes, such as cancer testis antigen (CTA), can also promote *RAD51* filament formation and simultaneously enhance the sensitivity to DNA damaging agents (Nichols et al., 2018). Dong et al. (2019) revealed that *RAD51* plays an important role in radiation reactions. The expression of *RAD51* is positively regulated by speckle-type POZ protein (*SPOP*), while ionizing radiation can lead to the downregulation of *SPOP* and influence the DNA damage response (DDR) pathway through *RAD51* (Dong et al., 2019).

The *KIF* gene family, which encodes proteins, is involved in many important physiological processes, especially intracellular transport, chromosome separation, mitotic spindle formation and cytokinesis. Some studies have suggested that mutations in the *KIF* gene family are involved in the formation of many cancers (Sheng et al., 2018; Xia et al., 2018; Xie et al., 2018). There are few studies on *KIF18b* and LUAD, and only Zhang L. et al. (2019) confirmed that LUAD patients with high *KIF18b* expression have a poor prognosis. Itzel et al. (2015) identified the regulatory role of *KIF18* in hepatocellular carcinoma (HCC) by performing an oncogenic microarray meta-analysis. *In vitro* experiments also confirmed that the downregulation of *KIF18B* could induce G1 phase arrest of the cell cycle and inhibit the proliferation, migration and invasion of cervical cancer cells, while its overexpression could promote the proliferation, migration and invasion of cervical cancer cells (Wu et al., 2018).

*KIFC1* is a c-type terminal kinesin that plays an indispensable role in the centrosomal aggregation of tumor cells (Farina et al., 2013). Using RT-qPCR and Western blot detection of NSCLC and adjacent normal lung tissue samples, Liu et al. (2016) found that *KIFC1* is highly expressed in NSCLC tissues and that silencing *KIFC1* inhibits NSCLC cell proliferation. Using flow cytometry to examine the cell cycle, we found that silencing *KIFC1* could arrest the cell cycle in G2/M phase, suggesting that *KIFC1* can be used as a biomarker for lung cancer diagnosis and treatment (Liu et al., 2016). In addition, Han et al. (2019) found that *KIFC1* is highly expressed in HCC and induces epithelial-mesenchymal transformation and HCC metastasis both *in vitro* and *in vivo*.

*FEN1* is an important component of the basal resection repair pathway of the DNA repair system and maintains genomic stability through DNA replication and repair (Shen et al., 2005). He et al. (2017) found that *FEN1* plays a key role in the rapid proliferation of NSCLC cells and confirmed in a mouse model that treatment with an *FEN1* inhibitor enhanced the sensitivity of NSCLC cells to DNA damaging agents and that combined therapy with cisplatin could significantly inhibit the progression of cancer cells. Using quantitative RT-PCR and immunohistochemical analysis, Zhang et al. (2018) revealed that *FEN1* is highly overexpressed in NSCLC tissues and that the higher the expression of *FEN1* is, the poorer the tumor differentiation and prognosis. *In vitro* experiments also confirmed that the downregulation of *FEN1* could lead to G1/S or G2/M cell cycle arrest in NSCLC cells and inhibit cell proliferation *in vitro* (Zhang et al., 2018). Therefore, inhibitors targeting *FEN1* may be a promising anticancer strategy.

*RAD54L* plays an important role in homologous recombination-related repair or DNA double-strand breakage (Rencic et al., 1996). There are few studies on this gene and NSCLC, especially the LUAD subtype, and only one report has indicated that it is highly expressed in NSCLC (Valk et al., 2010). Interestingly, two hub genes, including *RAD54L*, were shown to play an important role in the pathological mechanism of glioblastoma (GBM) in our study. Bai et al. (2018) confirmed experimentally in mice that *CHEK1* could induce the radioresistance of GBM cells by upregulating the expression of *RAD54L*, while *CHEK1* increased GBM cell apoptosis during radiotherapy by downregulating the expression of RAD54L.

Since NSCLC includes both LUAD and LUSC, their pathological mechanisms and immunoinfiltrating cells are different, so the therapeutic targets between the two may also be different (Faruki et al., 2017). It is interesting to note the WGCNA used to look for the hub gene that is correlated with miRNA, but the authors used samples from lung squamous cell carcinoma (LUSC) in the TCGA database and found another gene from the *KIF* family, *KIF15* (Qin et al., 2020). The two modules found in WGCNA were correlated with the enrichment scores of 28 types of immune cells, while the modules positively correlated with mRNAsi were negatively correlated with the enrichment scores, suggesting that mRNAsi-related genes may inhibit tumor immune cell infiltration. At the same time, as the expression level of the hub gene increases, the content of B cells and macrophages decreases. This finding suggests that these hub genes may be involved in tumor immunity, which warrants further research.

Our research has some limitations that should be mentioned. First, we used data from a public database to confirm our findings and did not perform further experiments to confirm the expression of related genes or research on the molecular mechanisms and pathways involved. Second, since our study examined data from a public database, the quality may not be guaranteed. At the same time, the results obtained by different chips may not be accurate due to differences between batches. Finally, most of the data we studied were obtained from the United States or Japan and are not representative of patients worldwide. Therefore, further well-designed biological studies with large sample sizes are needed to confirm our findings.

## Conclusion

*CHEK1, RAD51, KIF18B, KIFC1, FEN1*, and *RAD54L* may have a strong influence on LUAD stem cell maintenance. These hub genes may serve as control targets for LUAD CSCs, and further study of these genes may lead to the development of new anticancer therapies.

## Data Availability Statement

The datasets used and analyzed during the present study are available from the corresponding author on reasonable request.

## Author Contributions

YL conceived and designed the study, acquired and analyzed the data, and wrote the manuscript. YW and MC contributed to the data analysis and manuscript drafting. CH and XF designed the study and revised the manuscript.

## Conflict of Interest

The authors declare that the research was conducted in the absence of any commercial or financial relationships that could be construed as a potential conflict of interest.

## Footnotes

1 https://portal.gdc.cancer.gov

2 http://asia.ensembl.org/index.html

3 https://string-db.org/

4 https://cistrome.shinyapps.io/timer/

## Supplementary Material

FIGURE S1 Enrichment analysis of green and blue module. **(A)** GO analysis of green module, **(B)** GO analysis of blue module, **(C)** KEGG pathway enrichment analysis of green module. **(D)** KEGG pathway enrichment analysis of blue module. GO, gene ontology; KEGG, Kyoto Encyclopedia of Genes and Genomes.

FIGURE S2 Correlation between the gene module and ssGSEA scores of 28 immune cells, The correlation coefficient in each cell represented the correlation between the gene module and the scores, which decreased in size from red to blue. The corresponding *P*-value is also annotated. ssGSEA, Single simple gene set enrichment analysis.

FIGURE S3 Correlation of gene expression with immune infiltration level in LUAD. **(A)** *CHEK1*, **(B)** *RAD51*, **(C)** *KIF18B*, **(D)** *KIFC1*, **(E)** *FEN1*, **(F)** *RAD54L*. LUAD, lung adenocarcinoma.

TABLE S1 Clinical characteristics of GEO datasets.

## Abbreviations

AJCC: American Joint Committee on Cancer
ANOVA: analysis of variance
BP: biological process
CC: cellular component
CHEK1: checkpoint kinase 1
CSCs: cancer stem cells
DEGs: differentially expressed genes
EREG: epigenetically regulated
FC: fold change
FEN1: flap structure-specific endonuclease 1
GEO: gene expression omnibus
GO: gene ontology
GS: genetic significance
GSVA: gene set variation analysis
KEGG: Kyoto Encyclopedia of Genes and Genomes
KIF18B: Kinesin family member 18B
KIFC1: Kinesin family member C1
LUAD: lung adenocarcinoma
LUSC: lung squamous cell carcinoma
MCODE: molecular complex detection
MEs: module eigengenes
MF: molecular function
MM: module membership
mRNAsi: mRNA expression-based stemness index
MS: module significance
NSCLC: non-small-cell lung cancer
OS: overall survival
PFS: progression-free survival
PPI: protein-protein interaction
RAD51: recombination protein A 51
RAD54L: DNA repair and recombination protein RAD54-like
ROC: receiver operating characteristic
ssGSEA: Single simple gene set enrichment analysis
TCGA: The Cancer Genome Atlas
TIICs: tumor immune-infiltrating cells
TNM: tumor node metastasis
WGCNA: weighted gene coexpression network analysis.
