# Supplementary figures and images for: Weighted Gene Coexpression Network Analysis of Features That Control Cancer Stem Cells Reveals Prognostic Biomarkers in Lung Adenocarcinoma
Source: Front Genet. 2020 Apr 22;11:311. doi: 10.3389/fgene.2020.00311 (PMC7192063; doi:10.3389/fgene.2020.00311)

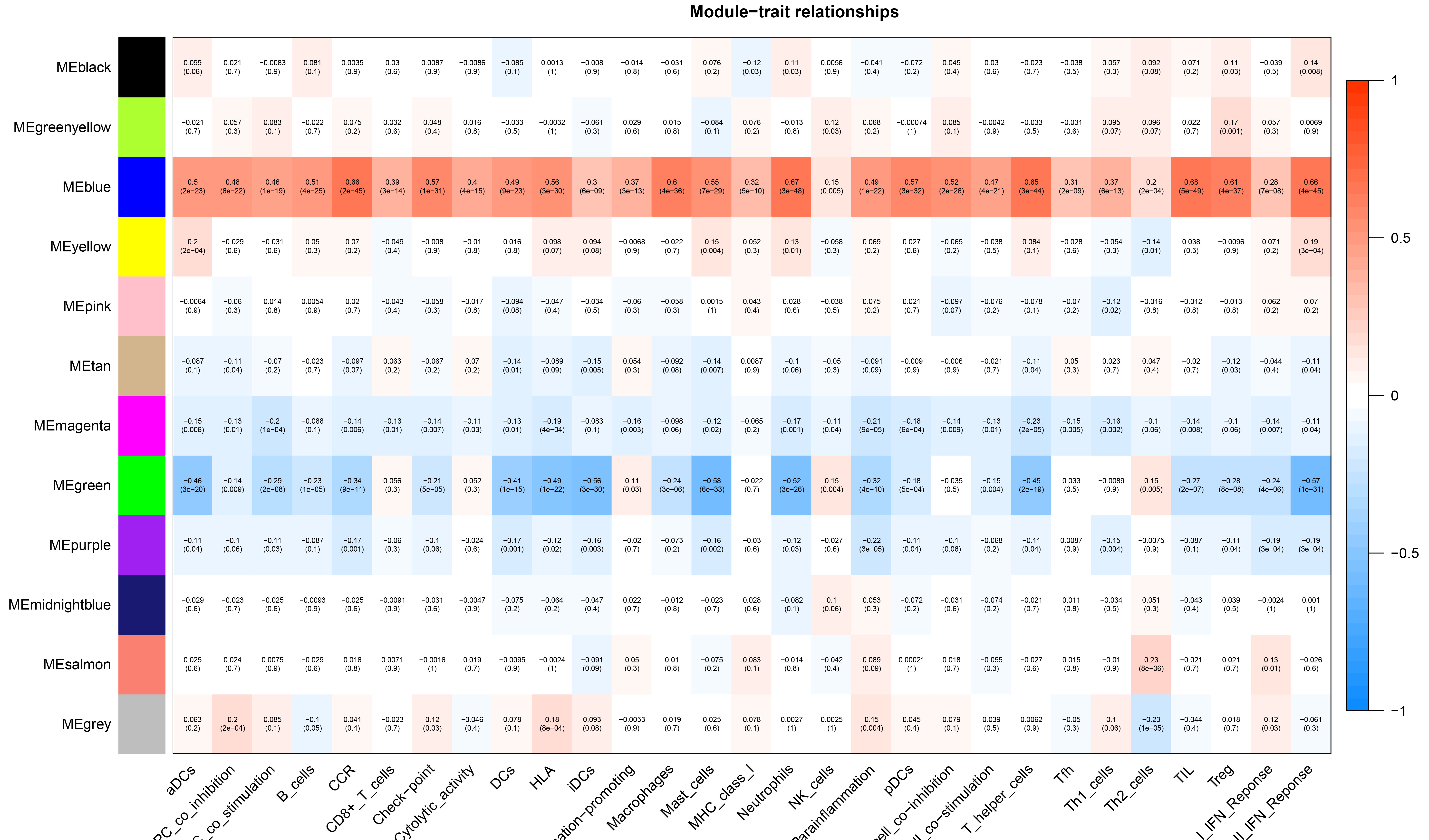

Supplement: FIGURE S1 — Enrichment analysis of green and blue module. (A) GO analysis of green module, (B) GO analysis of blue module, (C) KEGG pathway enrichment analysis of green module. (D) KEGG pathway enrichment analysis of blue module. GO, gene ontology; KEGG, Kyoto Encyclopedia of Genes and Genomes. [file Image_1.JPEG]
