# Supplementary material for: Weighted Gene Coexpression Network Analysis of Features That Control Cancer Stem Cells Reveals Prognostic Biomarkers in Lung Adenocarcinoma
Source: Front Genet. 2020 Apr 22;11:311. doi: 10.3389/fgene.2020.00311 (PMC7192063; doi:10.3389/fgene.2020.00311)
Supplement: TABLE S1 — Clinical characteristics of GEO datasets. [file Table_1.DOC]

| Record | Age | Sex (Male/Female) | AJCC stage (I/II/III/IV) | Grade (1/2/3) | Substype(Bronchioid/Magnoid/Squamoid) | Number of death (%) | Number of recurrence (%) |
| --- | --- | --- | --- | --- | --- | --- | --- |
| GSE13213 | 61.00±10.16 | 60/57 | 79/13/25/0 | - | - | 49 (41.9) | 58 (49.5) |
| GSE31210 | 59.58±17.40 | 105/121 | 168/58/0/0 | - | - | 34 (15.0) | 76 (33.6) |
| GSE26939 | 65.00±10.88 | 53/63 | 66/19/19/2 | 14/58/44 | 47/40/29 | 66 (56.8) | - |
| GSE32867 | 70.00±9.10 | 13/45 | 34/11/12/1 | - | - | - | - |
| GSE41271 | 58.72±12.30 | 94/88 | 106/22/49/5 | - | - | 71 (39.0) | 74 (40.6) |
| GSE43458 | 63.00±7.88 | 44/36 | - | - | - | - | - |

**Supplementary Table 1**. Clinical characteristics of GEO datasets.
